# Of Monsters and Monkeys: Queering the Study of Genetic Chimerism in Primates

**DOI:** 10.1002/ajpa.70362

**Published:** 2026-10-05

**Authors:** Rachel Voyt

**Affiliations:** ^1^ Department of Anthropology University of Texas at Austin Austin Texas USA

**Keywords:** biological chimerism, callitrichids, queer monster theory

## Abstract

Scientific research is a political enterprise, and is therefore susceptible to cultural biases. With its central role in understanding humans as a species, primatology is particularly vulnerable to this dynamic, requiring researchers to carefully assess both the influences shaping their work and the implications of their results. Here, I engage in this practice by critically examining the study of chimerism in callitrichid primates, a prominent model taxon in studies of human health and evolution. Chimerism, which refers to cells derived from two or more zygotic lineages within a single organism, is a defining feature of callitrichid biology. Chimerism also challenges the boundaries by which many biological categories are defined. Using the lens of queer monster theory, I investigate the relationship between the study of callitrichid chimerism and the conceptualization of callitrichid and human bodies, with particular attention to the role of “queer” sex and reproduction. I first discuss the origins of chimerism within biological discourse, showing how the biological chimera inherited “unnatural” connotations that continue to shape scientific research. I then examine how similar monster‐making practices surface in studies of callitrichids, arguing that patterns of omission and claims of exceptionalism serve to manage chimerism as a categorical threat. Finally, I explore the promises that callitrichid chimeras present as monstrous informants. I show that callitrichid chimeras can be used not only to expose the cultural boundary‐work structuring sex and reproduction, but to invite research practices that treat categorical deviation not as abnormal, but as evidence that these categories must be changed.

## 1 Introduction

Primatology emerged within biological anthropology as a means of better understanding what it means to be human. Findings from primate research are therefore routinely used to explain human characteristics and behaviors, including sex and gender roles, power dynamics, and morality (e.g., Burkart et al. 2018; De Waal 2022; Watts 2010). Yet this entanglement between human and nonhuman primates also renders primatology particularly vulnerable to cultural bias, not only through the intrusion of social norms into research practice, but through the co‐option of research results to reinforce those same norms (Haraway 1989; Radhakrishna and Jamieson 2018; Rees 2007; Sperling 1991; Strier 1994).

This pattern of mutual reinforcement is especially pronounced around axes of queerness (Haraway 1989). I use “queer” here in the sense used in queer theory, which encompasses any departure from the norm, but particularly that which defies the binary definitions and proscriptions of patriarchal cis‐heterosexism (Benshoff 2020). Across historical and contemporary contexts, perceived threats to hegemonic boundaries of sex, reproduction, and kinship have repeatedly been cast as monstrous and used to justify violence and erasure (Dahl 2018; Malatino 2019). In the United States, for example, trans and nonbinary people are routinely framed as predatory and dangerous, while appeals to a purported “biological truth” of immutable binary sex (e.g., Executive Order No. 14168 2025) are invoked to legitimize unprecedented levels of anti‐LGBTQ+ policy (Gwenffrewi 2022; Quinan 2025; Robinson 2023). In primatology, these dynamics become visible both in how studies are framed and in what studies are missing. Same‐sex sexual behavior, for instance, was historically framed as pathological and has largely been underreported or misrepresented to conform to heteronormative expectations (Anderson et al. 2024; Cunningham and Benítez 2024). Similarly, lemurs—a taxon that challenges sex‐associated norms on multiple counts—have been described as exhibiting a “syndrome” (e.g., Kappeler and Fichtel 2015; Kappeler and Schäffler 2008) and are frequently excluded from comparative analyses as a result (Lewis 2018). Together, these trends yield distorted representations of nonhuman primates and, by extension, biased reference points for understanding human biology and behavior.

If biological anthropologists are to avoid perpetuating this cycle, addressing the sociopolitical contexts shaping scientific knowledge production must become a nonnegotiable component of the research process. Queer monster theory offers a framework for doing so by approaching queer feminist analyses through the lens of monstrosity. Here, the “monstrous” is what unsettles our sense of order, producing epistemic vertigo by calling our worldview into question and revealing the failures of our systems of categorization (Mittman 2012). From the standpoint of those enforcing these systems, the monster represents a threat, something to be feared and eradicated (Carroll 2020). For those occupying the liminal spaces of these categories, however, monsters can also represent a promise: to expose the illusions of our boundary‐making practices while opening the possibility of more inclusive futures (Haraway 1992; Hellstrand et al. 2018). Queer monster theory embraces this dissonance by asking what monsters are, where they come from, and what they mean in order to expose and disrupt the boundary‐work through which culturally contingent categories are normalized as fact (Weinstock 2020).

In this article, I apply queer monster theory to the study of chimerism in callitrichid primates. This taxon comprises 52 species of marmosets, tamarins, lion tamarins, and callimicos distributed across the tropical forests of Central and South America (IUCN 2025). Callitrichids are notable among nonhuman primates for their prominent role in biomedical and comparative evolutionary research, where they are commonly used as models for human behavior, neurobiology, reproductive physiology, and related domains (Marini et al. 2019; Miller et al. 2016; Mitchell and Leopold 2015; Saito 2015). As such, the categories through which callitrichid bodies are described and interpreted not only determine our understanding of callitrichids but also shape the comparative claims used to understand humans.

Callitrichids are equally notable as chimeras, a term referring to organisms whose cells are derived from two or more distinct zygotic lineages (Anderson et al. 1951). Among mammals, small numbers of chimeric cells (i.e., “microchimerism”) are thought to be present in all placental species (Gammill and Harrington 2017; Nelson 2012). In callitrichids, however, chimerism routinely occurs on a much larger scale. Within this taxon, chimerism occurs in conjunction with the production of fraternal twins and other multiples, which is the typical birth pattern for all but callimicos (Ah‐King and Tullberg 2000; Harris et al. 2014). During embryonic development, littermates are connected via an interplacental vascular network that facilitates reciprocal transfer of hematopoietic stem cells, which persist, differentiate, and proliferate within the recipient throughout their lives (Benirschke et al. 1962; Wislocki 1939). Each callitrichid thus exists as a chimera of their “self” and their littermate(s). As such, callitrichids challenge the idea of the “individual” as a singular bounded entity, and subsequently destabilize the biological categories (e.g., sex, reproduction, and kinship) that depend on this conceptualization as an assumed foundation. Considering callitrichid chimerism through the lens of queer monster theory thus presents an opportunity to reassess the validity and usefulness of these categories, both by revealing the influence of categorical monsters created within human culture and by showing how these monsters are perpetuated through scientific research.

In treating callitrichid chimerism as a site of monstrous engagement, I begin with a brief genealogy of the chimera in biological research and identify the monsters constructed around it as a scientific category. I then examine the study of chimerism in callitrichids specifically, investigating the potential influence of historical and contemporary monster‐making practices. Finally, I explore how callitrichid chimerism might be used to unsettle conceptualizations of sex and reproduction within primatology and biological anthropology more broadly, opening space for alternative, more inclusive ways of knowing.

## 2 History of the Biological Chimera

The chimera entered biological discourse already burdened with a history of monstrosity. Long before it was formalized as a scientific category, the Chimera of Greek mythology functioned as a figure of impurity and threat, defined by the collapse of categorical boundaries across species and sex (Figure 1, B. Cohen 2010). Early 20th‐century biologists invoked these composite elements by adopting the term “chimera” to describe experimentally created interspecies mixtures in plants (Winkler 1907) and later in animal embryos (Spemann 1921). Early biological chimeras were thus marked as monstrous through both their multispecies composition and their emergence through “unnatural” technological intervention.

**FIGURE 1:**
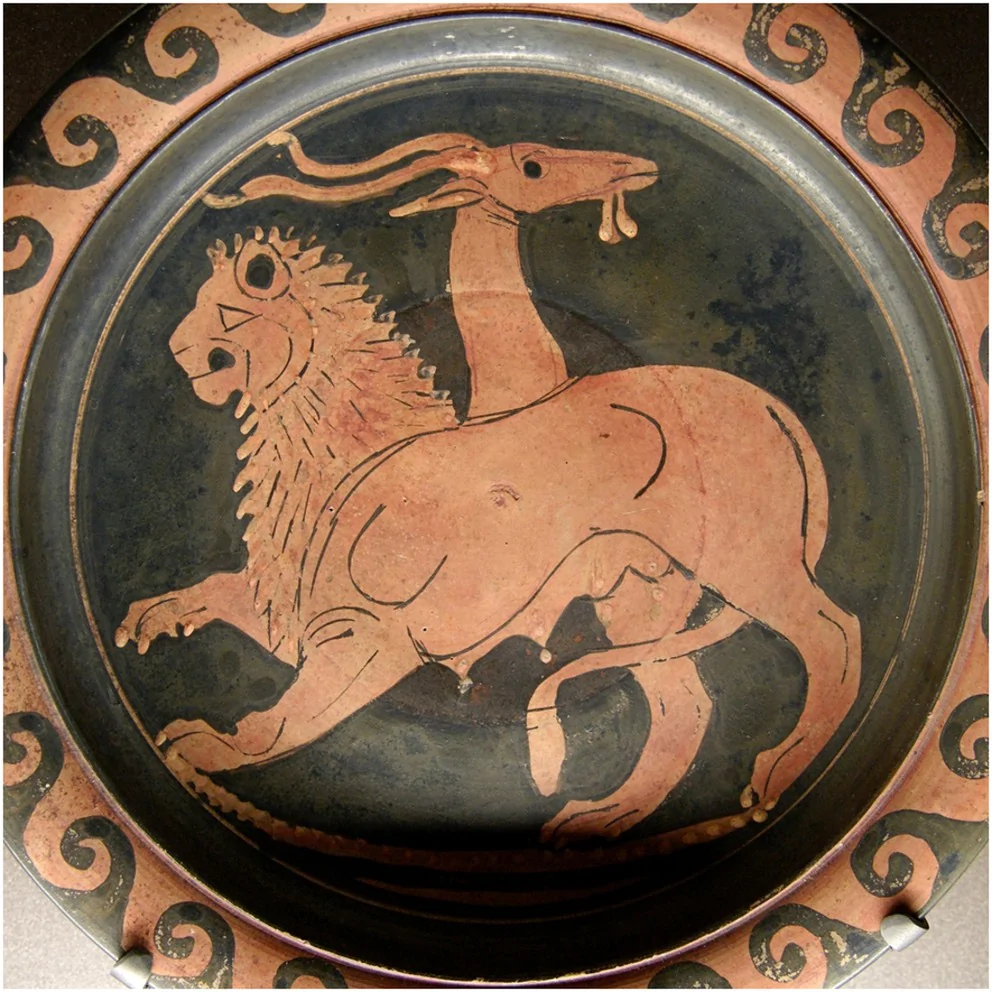
The Chimera of Greek mythology. Apulian red‐figure dish, ca. 350–340 bc. Louvre Museum, Public domain, via Wikimedia Commons.

These connotations persisted even after biological chimerism was found to occur outside the laboratory. When chimerism was first identified in humans through the discovery of two typically incompatible blood groups in a single individual (“Mrs. McK”), the rhetoric of monstrosity was amplified (Martin 2007, 2015). Correspondence between researchers shows that Mrs. McK was labeled a chimera specifically to highlight the “unnatural juxtapositions” she embodied (Owen 1953). Here, however, the biological chimera was not considered “unnatural” due to human tampering. Rather, this “unnatural” classification reflects nature as a cultural logic, in which nature does not stand for what *is*, but what *ought to be* within a given sociopolitical order (Mill 1874). By upsetting that order, the “unnatural” becomes what ought *not* to be, providing justification for tactics of social control, dehumanization, and monsterization (Barad 2011; Cohen 1996; Malatino 2019). The naturally occurring human chimera (and thus, the primate chimera) is therefore presented as unnatural because she violates the cultural understanding of what ought to constitute a human body, invoking the monstrous as a result.

In the decades following the case of Mrs. McK, naturally occurring chimerism has since been documented across myriad multicellular organisms through a variety of processes (Madan 2020; Rinkevich and Goulet 2025). In becoming recognized as more commonplace, the monstrous elements of chimerism might be expected to fade; monstrosity, after all, dwells at the gates of difference (Cohen 1996). In humans, however, chimerism has yet to shed these connotations. Microchimerism resulting from low‐level interplacental cell exchange between a developing fetus and the gestational parent, for example, is widespread in humans and likely in other placental mammals as well (Gammill and Harrington 2017; Nelson 2012). Even so, microchimeric cells are frequently framed as foreign entities breaching the border between self and other, and research predominantly focuses on their potential pathological effects (Kelly 2012; Nelson 2012). Less common forms of chimerism, such as those arising from interplacental exchange between twins or other multiples (“twin chimerism”) or from gametic fusion (“fusion chimerism”), become sites of monsterization even more readily. In these cases, chimeric cells are more prevalent and more likely to generate phenotypes that destabilize naturalized assumptions about sex, reproduction, and kinship. In other words, chimerism is more likely to be read as *queer*. Large‐scale chimerism resulting from mixed‐sex tetragametic fusion, for instance, can lead to visibly intersex traits (Wenk 2018); in threatening the claim to a binary sex, intersex people have been routinely pathologized and subjected to medical violence (Malatino 2019; Reis 2021). Large‐scale chimerism is also more likely to result in bodies that deviate from the genetic “rules” used to legitimize heteronormative understandings of reproduction and kinship, in which there exists one “real” mother and one “real” father whose roles are defined exclusively by their distinct genetic contributions (Hird 2004). Such deviations have resulted in chimeric parents being accused of fraudulently claiming children who were not “their own” (Norton and Zehner 2008; Sheets et al. 2018).

Taken together, these cases suggest that human chimerism remains difficult to disentangle from monstrosity not because it is intrinsically abnormal, but because it repeatedly activates axes of queerness. The chimera thus acts as a monstrous informant, illuminating not only the boundaries of biological categories but how those boundaries are weaponized to separate the “normal” from the queer. It is against this backdrop that nonhuman primates become an especially instructive site for tracing how such boundaries are reproduced, resisted, and potentially reconfigured.

## 3 Chimerism in Callitrichids

In callitrichid primates, twin chimerism is regarded as a defining characteristic rather than an anomaly and is thought to occur in all callitrichid species aside from callimicos (Ah‐King and Tullberg 2000; Harris et al. 2014). Within individuals, chimeric cells acquired via interplacental exchange between littermates have been found to comprise anywhere from 13% to 95% of those in hematopoietic tissues such as blood, bone marrow, and lymph nodes (Sweeney et al. 2012; Voyt 2024; Worley et al. 2014). If in callitrichids, as in humans, the majority of nucleated cells in the body are hematopoietic in origin (Hatton et al. 2023), chimeric DNA may therefore represent a substantial proportion of an individual's total DNA.

Despite the prevalence of chimerism at both taxonomic and individual levels, however, variation in its extent and expression across callitrichid species remains poorly characterized. Of the few studies conducted since callitrichid chimerism was described in the 1930s (Wislocki 1932, 1939), most focus exclusively on mixed‐sex twin pairs from captive populations of either the common marmoset ( *Callithrix jacchus* ) or cotton‐top tamarin (*Oedipomidas oedipus*), and only one study has examined chimerism in wild callitrichids to date (Voyt 2024). No studies have yet investigated microchimerism in callitrichids. Callitrichid genetic analyses also often avoid chimerism by relying on tissues such as hair that are less likely to contain chimeric DNA (e.g., Stendahl et al. 2025; Voyt 2024). Recent work, however, demonstrates that excluding chimerism and other species‐specific traits can significantly bias models of population history (Soni et al. 2025), findings that likely extend to other aspects of callitrichid research as well.

This paucity of callitrichid chimerism research is especially conspicuous given the importance of callitrichids as research models for human health and evolution. Concern about this gap has only recently prompted national agencies to call for further research on the implications of chimerism within these contexts (National Institutes of Health 2020). Notably, this call explicitly states that twin chimerism is “unique to marmosets and other callitrichid primates.” While callitrichids are indeed *distinctive* in that all individuals from litter‐bearing species are thought to be twin chimeras (Ah‐King and Tullberg 2000; Harris et al. 2014), this phenomenon is not *unique* to these species, as this phrasing implies. First identified in the case of Mrs. McK, twin chimerism in humans is estimated to occur in at least 8% of dizygotic twins and 21% of triplets (van Dijk et al. 1996). Though these percentages are small, they can nevertheless translate into a substantial number of individuals given the global frequency of twinning and multiple births (Monden et al. 2021). For humans with twin chimerism, callitrichids may thus offer an unusually informative comparative system. For the broader population, chimerism does not necessarily disqualify callitrichids as models, but it does require researchers to explicitly state which traits are being modeled, which are excluded, and why.

Read through queer monster theory, these patterns of omission and claims of uniqueness function as a form of boundary‐work, an attempt to contain a phenomenon capable of undoing the categories through which human and nonhuman bodies are made legible. As a monstrous figure, however, chimerism is unlikely to remain within these confines; as queer monster theory tells us, the monster always escapes (Cohen 1996). In what follows, I explore how callitrichid chimeras can take up this freedom as figures of promise—the promise of monsters to represent hope for a future beyond the margins (Haraway 1992) and the promise of nonhuman primates to aid in dismantling our inherited assumptions so that we might truly understand what it means to be human (Washburn 1973).

## 4 The Promises of Monsters and Monkeys

In exploring the promise of callitrichid chimeras, I turn from examining how human monster‐making practices have shaped the overall study of this phenomenon to how callitrichid chimeras constitute queer monstrous informants in their own right. I focus specifically on how callitrichid chimeras destabilize categories of sex and reproduction within primatology, including the kinship logics and evolutionary frameworks these categories often support. Their promise, in turn, can be discovered through a shift in perspective: to view callitrichid chimeras not as outliers within our current systems of categorization, but as a guide in the broader efforts toward determining how those systems might be reimagined (e.g., Ah‐King 2009; Beech and Novick 2023; Dahl 2018; Haraway 2008; Shildrick 2025; Velocci 2024).

Sex is widely recognized among scientists as an object of multiplicity, encompassing a suite of individually variable traits such as gamete production, chromosome configuration, morphology, physiology, and behavior (McLaughlin et al. 2023; Velocci 2024). Yet in primatology, as in other fields, sex is often treated as a strict binary, with multiple traits assumed to map cleanly onto essentialized categories of “female” or “male” on the basis of a single (and typically unspecified) proxy (Velocci 2024). Callitrichid chimerism underscores the limits of this practice. In callitrichids, individuals from mixed‐sex litters carry both XX and XY cell lineages in hematopoietic tissues (Voyt 2024; Wedi et al. 2017), and possibly in the germline as well (Ross et al. 2007). Littermates of any sex may also acquire microchimeric cell lines with different sex chromosomes via fetal‐parental cell exchange, as seen in humans (Bianchi et al. 1996; Johnson et al. 2021). As obligate chimeras, callitrichids therefore resist chromosomal definitions of sex and disrupt assumed correlations between chromosomes and other sex‐associated traits. Moreover, like humans, callitrichids can exhibit other intersex traits, such as variation in genital morphology (e.g., Sanchez‐Morgado et al. 2003; Wedi et al. 2011), further demonstrating that sex is not reliably captured by a binary classification.

Callitrichid chimerism also unsettles conventional narratives of reproduction. In both primatology and evolutionary theory more broadly, reproduction often functions as a biological imperative as well as the primary basis for inferring fitness. In turn, fitness is used to infer the potential for selection, though what fitness means and how it should be operationalized has been subject to ongoing debate (e.g., Ariew and Lewontin 2004; Bouchard 2011; Dobzhansky 1955; Hendry et al. 2018). Within primatology, fitness metrics typically treat the individual as the unit of interest, with fitness represented by the number of offspring produced relative to others in the study population. These metrics typically rely on estimates of genetic relatedness and kinship to identify potential mothers, fathers, and sometimes extended kin. In addition to disrupting binary conceptualizations of sex, chimeras further complicate fitness metrics by destabilizing the coherence of the overall “individual,” including the assumption that reproduction reliably transmits an individual's own genetic material (Rinkevich 2011). If callitrichid chimerism extends to the germline, for example, reproduction could transmit not an individual's “own” genetic material but that of a sibling (Ross et al. 2007). Furthermore, even if chimerism is limited to hematopoietic tissues, chimeric cells acquired from littermates may still be transferred to offspring as microchimerism (Figure 2), potentially including microchimeric cells from older siblings and even grandparents, as has been found to occur in humans (Gammill and Harrington 2017; Nelson 2012). Callitrichids thus emerge not as genetically homogeneous “individuals,” but as composite organisms whose reproduction is constituted across multiple bodies and timescales.

**FIGURE 2:**
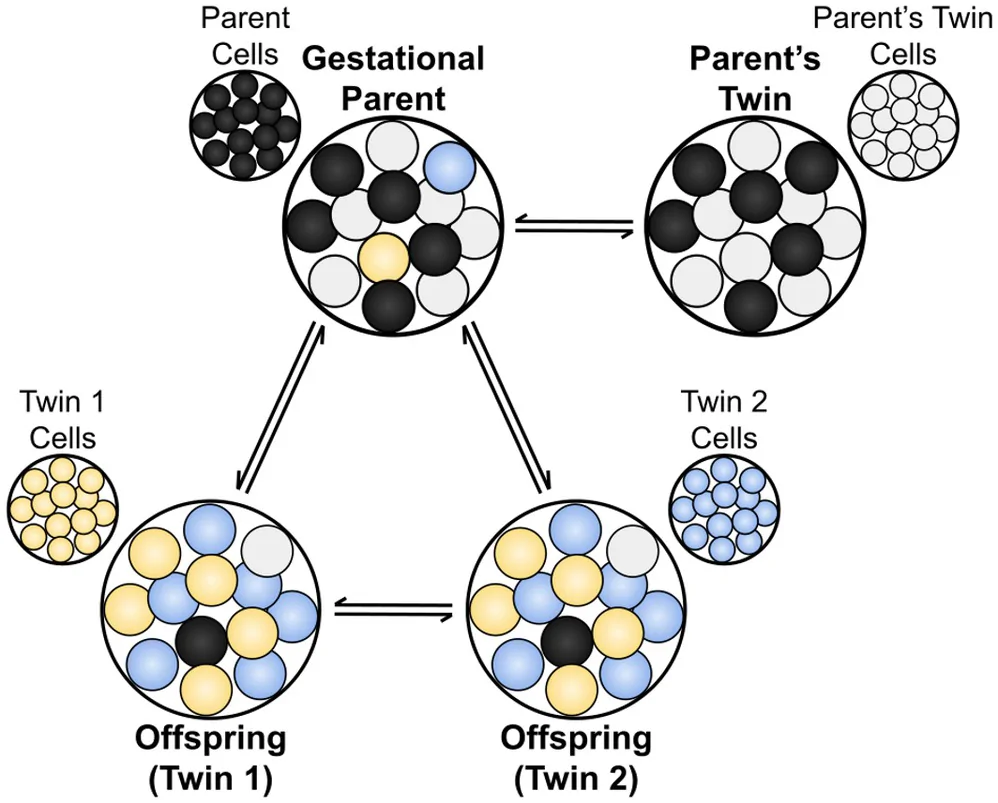
Sources of hematopoietic chimerism in callitrichid primates. Hematopoietic tissues in callitrichids are chimeric due to the exchange of hematopoietic stem cells between littermates in utero. These tissues may also contain microchimeric cells exchanged between the gestational parent and fetus, potentially including cells from the parent's littermates.

These boundaries of sex and reproduction break down further when chimerism is considered alongside other callitrichid reproductive adaptations. Callitrichids, for instance, exhibit a pronounced degree of skew in female reproductive success. Within callitrichid social groups, reproduction is typically limited to a primary breeding female across a multiyear tenure (Terborgh and Goldizen 1985), and some females may never give birth to surviving offspring in their lifetime (Goldizen 1996). Callitrichids are also cooperative breeders, a system in which alloparents routinely assist with offspring care regardless of kinship status (Garber 1997; Erb and Porter 2017). Some have suggested that chimerism may help to explain both female reproductive skew and cooperative breeding in terms of inclusive fitness (Haig 1999; Ross et al. 2007; Stephens 1989). The reasoning here is that chimerism increases the amount of genetic material shared between individuals, so any direct fitness benefits lost by not having offspring of one's “own” may be offset by indirect fitness benefits gained through alloparental offspring care. However, inclusive fitness still treats fitness as an individual property that presumes a stable distinction between the bodily material belonging to the “self” and to “others” (Folse and Roughgarden 2010; Hamilton 1964). Not only does chimerism muddy this self/other distinction, but in doing so it complicates designations of “alloparent” versus “parent,” “mother” versus “father,” as well as any other attempt to categorize kin relations or reproductive patterns. Consider the case of callitrichid offspring, for example, who inherit genetic material not from their mother, but from their mother's male twin (Ross et al. 2007). Consider also the cases in which the offspring in a litter have multiple genetic fathers (Voyt 2024), or the cases where offspring are nursed by a group member other than their mother (Smith et al. 2001; Watsa et al. 2017). Such nuances are ill‐captured by the traditional frameworks of sex and reproduction within primatology, signaling a need to reconsider the usefulness of these concepts as well as the interpretations we draw through their application.

Fully realizing the promise of callitrichid chimeras relies on a willingness to explore alternative ways of understanding. New scientific paradigms already exist, for example, that recognize the multiplicity in sexed physiologies (e.g., Massa et al. 2023). Likewise, frameworks have been proposed that treat chimeric reproduction and fitness as group‐level rather than individual‐level properties (Folse and Roughgarden 2010; Rinkevich 2019). Callitrichids offer a testing ground for these alternative approaches, with the potential to reshape not only how sex and reproduction are understood within primatology, but the comparative claims about human biology and behavior these categories are used to support.

## 5 Concluding Remarks

This article demonstrates how queer monster theory can illuminate the cultural boundary‐work that stabilizes biological categories of sex and reproduction as well as the kinship claims that often follow. Read as monstrous informants, callitrichid chimeras make visible how “monstrosity” is employed to police these categories in human and nonhuman primates alike, as well as what is at stake when that policing is normalized. When primatology defaults to inherited assumptions about sex and reproduction, it risks not only mischaracterizing its study species but lending scientific authority to the broader cultural machinery of queer monsterization and erasure.

The promises of monsters and monkeys therefore carry epistemic and ethical obligations. Meeting them is, in a sense, a monstrous practice; it requires connections and conversations across subfields and sustained attention to the entanglements of science and sociopolitical dynamics. Yet these obligations can also begin with small methodological choices: specifying the traits being measured rather than using “sex” as a silent binary proxy; defining and justifying what counts as “reproduction” and “fitness” within a given analysis; and acknowledging nonbinary sex and nonreproductive individuals without casting them as aberrant or without value. Practiced in these ways, primatology and biological anthropology can begin to unsettle naturalized bias rather than reproduce it.

Seen through this lens, the chimera returns us to the etymological origins of “monster,” from the Latin *monstrare*, “to reveal,” and *monere*, “to portend” (Beal 2002; Hellstrand et al. 2018; Weinstock 2020). Callitrichid chimeras expose the cultural underpinnings of the categorical systems used to make bodies legible, and they warn of what follows when those systems are upheld as immutable truths. By engaging with these monstrous aspects, we have the opportunity to treat callitrichids and humans not as entities transgressing boundaries, but as evidence that these boundaries should be dismantled. This is the promise of monsters, after all: to disrupt, to shift, to “queer” (Haraway 1992; Hellstrand et al. 2018).

What I really wanted to say was that a monster is not such a terrible thing to be (…) To be a monster is to be a hybrid signal, a lighthouse: both shelter and warning at once. (Vuong 2017)

## Author Contributions

**Rachel Voyt:** conceptualization, writing – original draft, writing – review and editing.

## Conflicts of Interest

The author declares no conflicts of interest.

## Data Availability

Data sharing not applicable to this article as no datasets were generated or analyzed during the current study.
